# mRNA Technology and Mucosal Immunization

**DOI:** 10.3390/vaccines12060670

**Published:** 2024-06-17

**Authors:** Antonio Toniolo, Giuseppe Maccari, Giovanni Camussi

**Affiliations:** 1 Global Virus Network, University of Insubria Medical School, 21100 Varese, Italy; 2 Data Science for Health (DaScH) Lab, Fondazione Toscana Life Sciences, 53100 Siena, Italy; g.maccari@toscanalifesciences.org; 3 Department of Medical Science, University of Turin, A.O.U. Città della Salute e della Scienza di Torino, 10126 Turin, Italy; giovanni.camussi@unito.it

**Keywords:** mRNA vaccines, mRNA constructs, systemic immunity, mucosal immunity, combined vaccination, extracellular vesicles, plant-derived extracellular vesicles

## Abstract

Current mRNA vaccines are mainly administered via intramuscular injection, which induces good systemic immunity but limited mucosal immunity. Achieving mucosal immunity through mRNA vaccination could diminish pathogen replication at the entry site and reduce interhuman transmission. However, delivering mRNA vaccines to mucosae faces challenges like mRNA degradation, poor entry into cells, and reactogenicity. Encapsulating mRNA in extracellular vesicles may protect the mRNA and reduce reactogenicity, making mucosal mRNA vaccines possible. Plant-derived extracellular vesicles from edible fruits have been investigated as mRNA carriers. Studies in animals show that mRNA vehiculated in orange-derived extracellular vesicles can elicit both systemic and mucosal immune responses when administered by the oral, nasal, or intramuscular routes. Once lyophilized, these products show remarkable stability. The optimization of mRNA to improve translation efficiency, immunogenicity, reactogenicity, and stability can be obtained through adjustments of the 5′cap region, poly-A tail, codons selection, and the use of nucleoside analogues. Recent studies have also proposed self-amplifying RNA vaccines containing an RNA polymerase as well as circular mRNA constructs. Data from parenterally primed animals demonstrate the efficacy of nasal immunization with non-adjuvanted protein, and studies in humans indicate that the combination of a parenteral vaccine with the natural exposure of mucosae to the same antigen provides protection and reduces transmission. Hence, mucosal mRNA vaccination would be beneficial at least in organisms pre-treated with parenteral vaccines. This practice could have wide applications for the treatment of infectious diseases.

## 1. Introduction

mRNA-based vaccines have emerged as a transformative force in immunization, offering a platform to protect against a variety of pathogens. By harnessing the cell’s natural machinery to produce proteins directly from mRNA molecules, the approach circumvents the need for replicating vectors (bacteria, fungi, and viruses), thus reducing biological risks during the production and delivery of vaccines as well as shortening development times. Their remarkable efficacy in addressing global health challenges emerged during the COVID-19 pandemic and is becoming a cornerstone of pharma technology. The 2023 Nobel Prize was awarded to Katalin Karikó and Drew Weissman for their finding that while the delivery of mRNA vaccines to mice incited substantial inflammatory responses through the activation of Toll-like receptors, the inoculation of mRNA that was modified by changing uridine with pseudouridine strongly abated reactogenicity [1,2]. The contributions of the two scientists have recently been summarized [3,4].

The mRNA platform may also be a basis for therapeutics, especially in the field of monoclonal antibodies (mAbs) [5]. Refinements are needed regarding the structure and production of mRNA constructs, the selection of mRNA packaging components to resist degradation and enter cells, and improved criteria for selecting administration routes and predicting the length of protection.

To date, mRNA vaccines have been administered predominantly via parenteral routes (intramuscular, intradermal, subcutaneous, intravenous, and into superficial lymph nodes) [6]. While these routes are effective in promoting resistance to systemic infection and in mitigating the risk of severe disease, they may fall short in addressing mucosal infections. In fact, it has been observed that—compared to natural infection—intramuscular COVID-19 mRNA vaccines provide much lower levels of mucosal immunity both in adults [7] and in children [8].

Actually, despite systemic immunity, microbes may continue to replicate at mucosal surfaces, evading local defenses and perpetuating interhuman transmission. For a wide range of infectious agents, mucosae serve as a critical interface both for establishing initial contact with the host and for exiting the body. Given the immune system’s inherent design to elicit a systemic response following superficial infections, leveraging mucosae for vaccination appears to be a strategy that is capable of triggering local immunity at the right point of pathogen entry. This would not only provide mucosal resistance that is critical to thwarting infection and interhuman transmission, but should also trigger a systemic response.

In general, vaccination programs need special consideration to another crucial aspect: the capacity to also be run in regions with limited medical infrastructure. Utilizing parenteral vaccination in such contexts may not be optimal due to the invasive nature of the practice and the need for trained personnel and sterile tools. In addition, the practice carries risks of infection, injuries by needles, and the occasional transmission of diseases other than the one the vaccine is intended to prevent. In contrast, mucosal vaccines may offer easy administration to the population and, usually, lower costs of production and delivery.

Developing and successfully testing mucosal vaccine formulations has been limited, primarily because of the scarcity of antigen delivery systems and of non-reactogenic mucosal adjuvants [9].

## 2. The Mucosal Immune System

In humans, the surface area of mucosae is over 100-fold that of skin. The concept of a “Common Mucosal System” emerged in the 1970s. Thus, the mucosal compartment represents a preeminent portion of the immune system. At different anatomical sites, mucosal epithelia have distinctive organizations, but the configuration of follicles, interfollicular regions, subepithelial dome regions, and follicle-associated epithelial cells is consistent [10]. Leukocytes residing in the lamina propria of mucosae include the intraepithelial lymphocytes, i.e., a population of cells located at the surface epithelium in an environment characterized by constant and diverse antigenic encounters with environmental and microbial constituents. Mucosal leukocytes comprise a variety of cell types with distinct innate, antigen-presenting, helper, effector, and regulatory properties. They include tissue-resident memory T cells, effector memory T cells, regulatory T cells, unconventional T cells such as mucosal-associated invariant T cells, natural killers, dendritic cells, and gamma-delta T cells [11,12].

The common mucosal system entails a network of interactions among leukocyte subsets that reside within the mucosa as well as interactions with those inhabiting other lymphoid compartments, such as lymph nodes, primary lymphatic organs, and circulating leukocytes. The communication of lymphoid cells with epithelial cells reinforces the mucosal barrier and needs to balance the induction of tolerance (e.g., versus food components and microbial commensals) with the ability to elicit an adequate protection against bacteria, fungi, parasites, and viruses. Mucosa-associated lymphoid cells share essential traits that include an epithelium-adapted profile, innate-like properties, cytotoxic potential, the ability to initiate immune responses to novel antigens, and the ability to produce cellular effectors of immune protection together with antibodies of the IgA class [13]. Though the different mucosal systems are interacting, it should be noted that some compartmentalization needs to be considered since nasal immunization mostly protects the respiratory tract and—while it is known that oral immunization is effective for salivary glands and parts of the intestine and mammary glands—is almost ineffective for the genital tract [14]. Thus, generalizations must be avoided.

In most cases, systemic vaccination protects people from disease but fails to generate a mucosal immune response capable of preventing the local replication of microbes and the interhuman transmission of respiratory, digestive, and urogenital pathogens. Here, we discuss whether mucosal vaccines may stimulate an immune response at the entry sites of pathogens but also a systemic response that is capable of protecting the body from microbial invasion.

So far, live attenuated vaccines have been the most effective tools for protecting against infection, disease development, and microbial transmission. Live vaccines also elicit protective responses of long duration and may provide herd immunity [15]. Could the mucosal administration of mRNA-based vaccines emulate the efficacy of self-replicating microbial agents? [16]. In principle, memory T cells are linked to immunosurveillance and the duration of immune protection. However, a quantitative estimate of these cells cannot be carried out in the mucosae of a living organism. Regarding mRNA vaccines, novel approaches are needed since inadequate results have been obtained using traditional technologies [17].

In humans, ethical and practical issues limit the methodology for evaluating the response to mucosal immunization. In short, vaccine trials of mucosal immunogens usually assess the following parameters: (a) the levels of secretory IgA (S-IgA) in relevant mucosae, (b) the levels of IgG, IgA, and IgM in serum, (c) and—as a proxy for mucosal immunity—the numbers of innate cells, helper/cytotoxic T cells, as well as B cells in peripheral blood using cytofluorimetry or ELISPOT [18]. In mucosae, innate immune cells play an early protective role against a wide range of pathogens and help to elicit de novo immune responses. T cells play crucial roles in clearing virus-infected cells and regulating B cell functions. Though cellular immunity represents a key indicator for mucosal vaccines, only a few studies of severe respiratory infections have investigated bronchoalveolar lavage fluids, which allow for adequate numbers of cells to be recovered. Most studies have only explored nasal secretions or saliva that contain small and unstable numbers of cells. Thus, obtaining representative mucosal samples to evaluate cellular immunity after vaccination poses significant challenges. Of note, in mucosal secretions, it is also difficult to measure specific antibody levels of different subclasses due to their tiny and variable concentrations.

In serum, IgA represents the second most abundant antibody after IgG. In humans, two major IgA subclasses are known: IgA1 has a longer hinge region that is absent in IgA2, making IgA2 more resistant to bacterial proteases. Different glycosylation patterns of IgA1 and IgA2 influence their functions, including their antigen binding and effector functions [19] as well as their pro-inflammatory or tolerogenic properties [20]. An additional difference between S-IgA in mucosal secretions and serum IgA is the dimeric or multimeric structure of S-IgA compared to the mainly monomeric forms present in serum. S-IgA is composed of two or more IgA molecules linked through a J-chain to the secretory component [21]. The dimeric/multimeric structure of these antibodies enhances their ability to bind large numbers of antigen molecules [22]. This translates into augmented efficacy. For instance, the virus-neutralizing activity of an IgA is higher compared to that of a corresponding IgG antibody [23]. The study of Sun and colleagues also shows that dimeric IgA may have an over 200-fold higher neutralizing potency compared to monomeric IgA. This study also suggests that plant-derived IgA could be used for the systemic or topical therapy/prevention of infections. Thus, together with other indicators, the evaluation of mucosal vaccines should include separate determinations for the two IgA subclasses both in mucosal secretions and in serum.

## 3. Mucosal Immunization

Studies in animals indicate that mucosal immunization cannot be achieved by simply repurposing mRNA vaccines as formulated for parenteral administration. First, the mucus layer and enzymes in secretions obstruct both the stability of mRNA cargoes and their entry into epithelia, microfold cells, and antigen-presenting cells. Second, the reactogenicity of mRNA vaccines—deemed acceptable for intramuscular delivery—may compromise their safety in mucosae due to the exquisite sensitivity of these sites to inflammatory stimuli [24]. A proper combination of non-stimulatory ionizable lipids combined with select Toll-like receptor (TLR) agonists or stimulator of interferon genes protein (STING) agonists could be effective in delivering mRNA into epithelia and immunocompetent mucosal cells [25]. In humans, the second dose of COVID-19 mRNA vaccines enhances humoral and cellular immunity but also induces more severe reactogenicity than the first dose [26]. This could be linked to the release of pro-inflammatory molecules causing reactogenicity that may also contribute to boost adaptive immunity. IFN-gamma is one of the possible mediators [27]. Identifying the innate immune pathways responsible for reactogenicity and immunogenicity will certainly benefit the advancement of mucosal vaccines.

Notably, recent studies demonstrate that the mucosal administration of SARS-CoV-2 vaccines to macaques that previously received parenteral vaccination induces a strong mucosal response in the respiratory tract, i.e., at the entry site of the pathogen. In fact, intratracheal Ad26 boosting provided near-complete protection that correlated best with mucosal humoral and cellular immune responses [28].

A study in humans [29] compared the serum IgG, saliva IgG, and saliva S-IgA responses in individuals who received COVID-19 mRNA booster vaccinations or who experienced breakthrough infections. It was found that intramuscular mRNA boosters could induce robust serum and saliva IgG responses, especially in individuals who had not experienced infections before, but the saliva S-IgA responses were weak. In contrast, breakthrough infections in individuals who had received a primary mRNA vaccination were followed by high levels of serum and saliva IgG as well as S-IgA. Individuals who had received a booster dose and then had a breakthrough infection showed low IgG induction in serum and saliva but still responded with high levels of saliva S-IgA. Taken together, the data suggest that exposure to an antigen at respiratory surfaces is an essential and effective stimulus for inducing mucosal S-IgA in the respiratory tract. Both studies indicate the possible strategy of combining systemic immunization with mucosal immunization. This approach could thwart the replication of a virus in the airways and limit its airborne transmission in the population. Over the past few years, attention has moved to the delivery of inhalable vaccines to lungs since the pulmonary route may generate a more robust immune response compared to the nasal route of administration [30,31]. To this purpose, dry powder vaccine formulations are to be preferred for multiple reasons, provided that the employed drying techniques are not detrimental to the chemical integrity of the vaccine and may yield particles with a proper size distribution of less than 5 μm [30]. Interest in mucosal immunization also stimulated the development of unique novel technologies. First, a nanoemulsion (NE) of lipids/virus protein plus an RNA-based RIG-1 agonist (IVT) as an adjuvant for nasal immunization against SARS-CoV-2 was developed [32]. The NE/IVT combination can activate the three major innate receptor classes (TLRs, RLRs, and NLRs) that are necessary for inducing antiviral immune responses. This work underscores the value of a mucosal boost vaccination to obtain local and systemic immune responses in previously vaccinated subjects. Second, the nasal administration—without adjuvants—of the ubiquitous and large *E. coli* bacteriophage T4 was engineered to express the selected virus protein [33]. Apparently, the sole administration of phage T4 was sufficient to induce a robust cellular and humoral antiviral response in the mucosa of experimental animals. Due to the ease of manufacture and structural stability, the phage T4 platform could provide a unique inexpensive opportunity for producing mucosal vaccines.

## 4. Extracellular Vesicles as Carriers of mRNA Vaccines

Extracellular vesicles (EVs) are small membrane-bound particles released by cells into the extracellular space and include exosomes and ectosomes/microvesicles. EVs are proper for most living systems, including animals and plants. They play a role in intercellular communication by transporting bioactive molecules, such as proteins, lipids, and nucleic acids [34].

EVs have the potential to be employed for mRNA vaccines thanks to their intrinsic properties [35,36], described below, that make them a valid alternative to parenteral administration:

(a) Natural Cell-to-Cell Communication: EVs are involved in normal physiological processes as mediators of cell-to-cell communication. Exploiting this natural communication system for vaccine delivery may be advantageous in terms of the body’s response to the introduced mRNA. EVs may also modulate immune responses [37,38].
(b) Protection of mRNA: EVs can protect the enclosed mRNA from degradation and immune system recognition, thus improving the stability and effectiveness of mRNA vaccines [35,37,39,40,41,42].
(c) Targeted Delivery: EVs can be engineered to express specific surface proteins that allow them to target specific cell types or tissues, thus enhancing the precision of vaccine delivery. Several methods have been developed to engineer EVs by modifying their surface with the purpose of targeting drug delivery [43,44]. For a systematic review, see the study by Raghav and colleagues [45].
(d) Reduced Reactogenicity: EVs may help reduce inflammatory reactions associated with mRNA vaccines, making them safer and more acceptable for therapeutic applications.
(e) Potential for Personalized Medicine: The ability to modify EVs for specific target cells unlocks the possibility for personalized medicine, better adapting vaccines to individuals’ unique needs [46,47].

While the concept is promising, the implementation of EVs as mRNA carriers requires intensive research. Challenges include optimizing production methods, ensuring the loading of mRNA into EVs, and addressing regulatory and safety considerations.

There are two different methods that have been proposed for loading EVs with drugs, nucleic acids, or other cargo. The main strategies are either manipulating the cells that make the EVs or directly modifying purified EVs [48,49,50,51].

With the first approach, researchers may load up cells with the desired molecule, and the cells will then make EVs containing the select cargo [52].

Cells can also be genetically modified to produce EVs that specifically carry the desired molecule. This may help in the delivery of drugs or genes. However, messing with genes raises safety issues that need to be addressed before using this approach in animals and humans. The remaining approach is to modify purified EVs [53]. Loading cargo into EVs may be carried out either passively or actively. For passive loading, EVs are incubated with high concentrations of the molecule, allowing it to passively diffuse into the EVs. This depends on variables such as water solubility or the charge of the select molecule [54]. This method does not damage the EV structure. Incorporating certain proteins capable of binding mRNAs on their surface may facilitate the loading of EVs [55,56]. A study identified Annexin A2 as a surface molecule on human serum EVs that is capable of binding exogenous RNAs, thus permitting the selective transfer of microRNAs [51,57]. Small interfering RNAs (siRNAs) conjugated with cholesterol, as well as hydrophobically modified siRNAs, have also been effectively loaded into EVs [58,59].

For active loading, multiple techniques have been proposed, including electroporation, osmotic shock, and ultrasound waves to temporarily make EV membranes more permeable, favoring the internalization of exogenous molecules [60,61]. Electroporation has been used for siRNA, miRNA, and DNA, but it can aggregate EVs or damage membranes. The membrane anchor technique using lipid conjugated nucleic acids seems more effective for siRNA [58]. Cationic transfection has also worked to load both siRNA and miRNA [62,63].

The efficiency of loading varies depending on the EV source and the cargo type. A limitation is that some techniques may disrupt EVs or deplete their native content upon loading exogenous molecules. Thus, a variety of approaches have been attempted to optimize EVs for targeted drug and gene delivery.

EVs have shown promise in vaccinology. EVs can carry antigens on their surfaces as well as immunostimulatory molecules that are capable of triggering both cell-mediated and humoral immunity [64,65]. For example, EVs containing tumor antigens interact with antigen-presenting cells to stimulate a CD8+ T cell response in mice vaccinated with EVs loaded with tumor antigens [66]. Antigens associated with EVs have proven to be more immunogenic in mice than soluble molecules.

Bacterial outer membrane vesicles have also been tested for vaccines. Their inflammatory nature, however, needs careful dosage and safety assessments [67]. Bacteria secrete EVs that play important roles in communication with the host and in pathogenesis [68]. For instance, EVs from H. pylori carry pathogenic factors [69]. Since bacterial EVs contain antigenic, toxic, and reactogenic constituents, they can stimulate host immunity by interacting with a variety of cells and activating both the innate and adaptive responses. As bacterial EVs are non-replicative, they may offer a way to immunize against bacteria without the infectious risk of live bacteria. Intranasal immunization with N. meningitidis EVs has induced effective mucosal immunity with the production of both IgG and S-IgA. EVs have also been shown to trigger both humoral and cell-mediated responses, an advantage over existing mucosal adjuvants. Genetically modified bacteria could produce EVs displaying neoantigens, thus serving both as adjuvants and immunogens [70]. The adjuvant activity of bacterial EVs relates to associated LPS, but LPS toxicity can be a limitation for clinical use. Therefore, balancing low LPS toxicity will be important. Another challenge is the low yield of EV production from most bacterial species. Current studies aim to optimize stress and temperature conditions for efficient EV secretion in non-pathogenic bacteria.

Little experience with oral mRNA vaccines has been presented so far, and mRNA instability represents a problem. The mucosal vaccines currently undergoing clinical trials mainly use protein antigens, live attenuated viruses, or viral vectors of immunogens. Formulating freeze-dried mRNA-loaded EVs could be an effective approach for oral vaccines [71,72].

As anticipated by Neutra and other investigators [73,74], there are pros and cons to oral vaccine administration. Improved compliance, easy storage, distribution logistics, and the induction of not just IgG but also IgA and T cell immune responses are pros. Over 90% of pathogens reach the body through the gastrointestinal tract, the urinary system, or the respiratory system. Therefore, the mucosal response is particularly important for preventing pathogen invasion. Oral vaccines also require less purification than injectable ones due to the non-sterile environment of the gastro-intestinal or respiratory tracts, which could allow for a significant reduction in manufacturing costs. The main challenges are related to vaccine degradation from gastric acidity, digestive enzymes, and bile salts. However, these obstacles may be at least partly overcome by formulation in capsules resistant to a low pH and enzyme degradation. In addition, oral vaccines usually require high and repeated doses of antigens, which could preferentially induce tolerance instead of immunization. Encouraged by collaboration with bioengineers, research has especially been focused on lipid nanoparticles that can bind and stabilize multiple immunogens, making them absorbable through the epithelial barrier [75]. Safety concerns might emerge, such as gluten hypersensitivity or related chronic intestinal disorders in genetically predisposed individuals [76].

In addition, it should be taken into consideration that antigen–antibody complexes are trapped by dendritic cells that activate adaptative responses [77]. One approach to induce mucosal immunity is through intranasal administration, even if vaccine contact with the olfactory bulb and the possible entry of vaccine components in the brain call for caution. For instance, cases of Bell’s palsy have been seen after intranasal vaccination with adjuvanted vaccines [78]. EVs loaded with SARS-CoV-2 S1 mRNA have been administered to mice and triggered secretory antibodies and T cell immune responses comparable to those obtained using oral immunization [79,80,81,82,83].

Wang et al. [81] investigated the intranasal administration of lung cell-derived EVs containing mRNA encoding the SARS-CoV-2 Spike protein. The vaccine consists of a recombinant SARS-CoV-2 receptor-binding domain conjugated to lung cell-derived EVs. Animal immunization elicited specific IgG and IgA antibodies and a T cell-mediated immune response. In hamsters, the lung cell-derived EVs were superior to a comparable liposome-based vaccine since it greatly reduced the severity of pneumonia caused by SARS-CoV-2. Notably, the lyophilized form of this mRNA vaccine was stable for at least three months [81]. Lung-derived EVs containing mRNA encoding the S protein of SARS-CoV-2 were also administered as inhalable powder both to rodents and non-human primates. The EV vaccine was superior to synthetic liposomes due to better biodistribution in the lungs [82,83].

A vaccine composed of EVs derived from the HEK293 cell line fused with lipid-coated mRNA encoding the S and N proteins of SARS-CoV-2 was developed by Tsai and colleagues [35]. mRNAs were coated with cationic lipids, and then the lipid-coated mRNAs were loaded into purified EVs or into lipid nanoparticles. mRNA-loaded EVs were characterized by efficient mRNA encapsulation (approximately 90%), a high mRNA content, a consistent size, and a polydispersity index below 0.2. When using mRNA encoding the red light-emitting luciferase Antares2, it was observed that mRNA-loaded EVs were superior to mRNA-loaded lipid nanoparticles for delivering functional mRNA into human cells in vitro [43]. In mice, the injection of Antares2 mRNA-loaded EVs also led to light emission following injection into the vitreous of the eye or into skeletal muscle. Additionally, it was shown that the repeated injection of Antares2 mRNA-loaded EVs drove sustained luciferase expression across six injections spanning at least 10 weeks without evidence of signal attenuation or adverse events at the site of injection. While mRNA loaded into synthetic nanoparticles was cytotoxic in vitro, mRNA loaded in EVs stimulated long-lasting immunity [43].

## 5. mRNA Vaccines Encapsulated into Extracellular Vesicles from Edible Plants

Since upscaling cultures of human cells to produce EVs is technically demanding and requires complex purification stages as well as difficult biosafety controls, alternative resources are being explored. EVs from edible plants closely resemble EVs of mammalian tissues [44]. They are constituted by a bilayer membrane and can be loaded with exogenous nucleic acids, including mRNA, regulatory small RNA, and DNA plasmids [46,84,85]. Plant-derived EVs are abundant in fruit juice, and their production is scalable. In addition, selected plants that have been a part of human diets for a long time are expected to be non-toxic and—likely—non-immunogenic.

**Figure 1:**
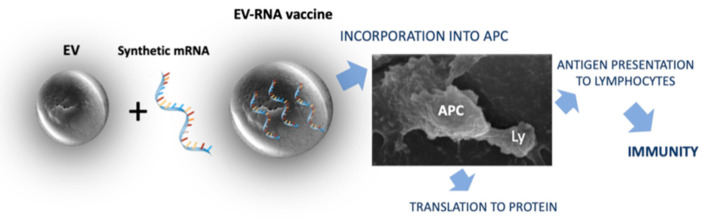
The activation of adaptive immunity by an mRNA construct encapsulated into extracellular vesicles (EVs). EVs protect the mRNA from degradation and allow for its penetration into mucosal epithelial cells and antigen-presenting cells (APCs). Here, it is translated into the encoded protein which, after processing, is displayed on the cell surface complexed with MHC molecules. The interaction of APCs with lymphocytes (Ly) allow them to recognize the foreign protein and become activated.

The general procedure for immunization using mRNA constructs loaded into plant-derived EVs is summarized in Figure 1.

Pomatto and colleagues [84,86] explored the use of plant-derived EVs obtained from orange fruits (Citrus sinensis) as carriers for SARS-CoV-2 mRNA vaccines. mRNAs were loaded into EVs utilizing a cation-based interaction combined with controlled osmotic stress to load mRNA molecules into EVs. By exploiting the negative charge of EVs, a positive linker allowed for interactions of negatively charged mRNA. Controlled osmotic stress allowed for mRNA flipping inside EVs. Using this technique, mRNA encoding for the Spike S1 subunit (S1), Full Spike (FS), and nucleocapsid (N) proteins was loaded into orange-derived EVs. The loading efficiency was 72% ± 11% for all studied mRNAs, and the loading capacity was 3.51 ± 1.09 ng per 10^11^ EVs. The reported values remained constant regardless of the mRNA length.

The potential of plant-derived EVs as a platform for administering mRNAs has been evaluated upon administration by the parenteral, oral, and intranasal routes. Accurate studies have demonstrated the integrity and stability of mRNA molecules once incorporated into orange EVs. MRNA protection was achieved due to their effective encasing by EVs, as permeabilization by Triton X-100 of the EV membrane abolished resistance to the ribonuclease.

As shown in in vitro experiments, mRNA was successfully delivered to antigen-presenting cells and activated T lymphocytes. EVs carrying S1- or FS-encoding proteins were administered to mice via gavage as a liquid formulation free of adjuvants. The results of oral immunization were compared to those of intramuscular administration. Oral or intramuscular vaccination with S1 or FS induced a comparable production of IgM and IgG antibodies in serum as well as virus-neutralizing antibodies. Interestingly, oral administration induced a significant production of virus-specific S-IgA [86]. A biodistribution study of orally administered EVs showed that part of the EVs were absorbed in the stomach instead of reaching the small intestine, where the majority of immune cells are located. With the aim of avoiding the absorption of EVs within the stomach, SARS-CoV-2 S1 mRNA-loaded EVs were inserted into small acid-resistant capsules. Capsules were administered by gavage to rats. Rats that were given lyophilized orange-derived EVs encapsulated in acid-resistant capsules produced virus-specific S-IgA and T lymphocyte responses [84]. Stability testing at 4 °C and 20 °C showed that lyophilized preparations of mRNA encapsulated into orange EVs remained functional for one year.

Thus, mRNA vaccines encapsulated into orange-derived EVs were effective through three routes of administration and triggered both humoral and cell-mediated responses. When formulated as lyophilized powder, these vaccines are stable and may be given through the oral route as well as through inhalation. Thermal stability and easy administration may be especially important for low-income countries.

## 6. Design of mRNA Constructs Encoding Antigenic Proteins

While mRNA vaccines offer numerous advantages, there are notable challenges associated with their delivery. Research is focused on developing improved delivery systems to safeguard mRNA vaccines and ensure their entry into cells [48]. Potential side effects, including heart, renal, and microvascular damage; blood clotting; as well as allergic reactions are possible with mRNA vaccines [87]. These have been traced, in part, to hypersensitivity reactions elicited by LNP-mRNA components, possibly PEGylated lipid nanoparticles [88]. An encapsulation rate of over 90% is desired. The rapid degradation of mRNA in the body after administration [89] or exaggerated cytokine responses [90] are critical obstacles in medical applications.

**Figure 2:**
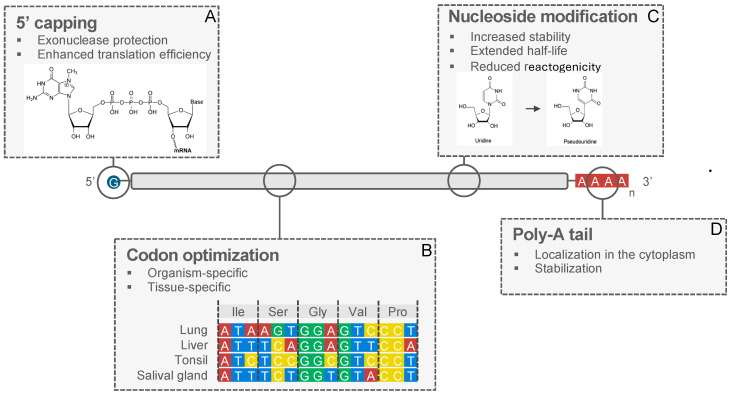
Enhancing mRNA properties through predictive design. (**A**) 5’ capping: improves molecule stability by shielding it from exonucleases. (**B**) Codon usage: influences tissue- or organ-specific expression by optimizing translation speed, based on availability of tRNAs. (**C**) Nucleoside modifications (e.g., replacing uridine with pseudouridine): enhances mRNA stability, reduces activation of innate immunity, improves translation efficiency. (**D**) 3’ poly-A tail: enhances mRNA stability and translation efficiency.

The translation of mRNA into functional proteins is contingent upon optimizing key elements within mRNA constructs. The stability and translation efficiency of mRNA sequences can be improved through modifications of the 5′ cap region, codon optimization, modifications of nucleosides, the 3′poly-A tail, and other means. Select aspects are summarized in Figure 2.

### 6.1. Five-Prime Cap Structure

All eukaryotic mRNA contains a 5′cap structure consisting of an N7-methylated guanosine (m7G) which is added during RNA post-transcription modifications. The 5′cap structure has a double role in the RNA life cycle: it initiates protein synthesis and it serves as a protective barrier against exonuclease cleavage, thereby enhancing mRNA stability. Notably, the cap structure aids the host’s discrimination between self and non-self mRNA molecules, underscoring its importance in host–pathogen interactions and reactogenicity [91].

The modification of m7G was introduced to improve mRNA translation. For example, replacing m7G with 7-benzylated guanosine significantly enhances translation efficiency [92], which is further boosted by attaching m7G with another m7G via tetraphosphate (m7Gppppm7G), resulting in analogs with higher translation efficiency compared to that of natural eukaryotic 5′caps. 

Additionally, synthetic modifications such as anti-reverse cap analogs (ARCAs) have been developed to further enhance mRNA stability and translation efficiency. ARCA-capped mRNA exhibits improved translation efficiency and prolonged half-life compared to conventional cap analogs, leading to enhanced protein expression in cultured cells [93].

A proprietary capping technique called CleanCap utilizes a fully enzymatic process to cap mRNA transcripts, avoiding the use of potentially immunogenic chemical reagents. This enzymatic approach results in high-quality, homogeneous mRNA with a precisely defined cap structure, ensuring optimal stability and translation efficiency [94]. CleanCap-capped mRNA is particularly well suited for applications requiring high purity and reproducibility, such as gene therapy and vaccines.

### 6.2. Three-Prime Poly (A) Tail

Poly(A) tail is an essential element in most protein-encoding RNA molecules, providing stability and improving translation efficiency [95]. Poly(A) tail is added to mRNA in the cell nucleus in a post-transcriptional process downstream of the gene-encoded polyadenylation signal (AATAAA) [96]. Eukaryotic transcripts usually receive a poly-A tail of approximately 200 nucleotides (nt) in mammals and 70 nt in yeasts [97]. For mRNA constructs, an optimal poly(A) tail length of approximately 100 nt is adequate to reduce RNA degradation [98]. Other approaches have been evaluated, including the adoption of segmented poly(A) sequences. This method involves the insertion of interspersed smaller spaces between poly(A) stretches and may improve the stability of plasmid DNA and the expression of mRNA in vivo [99,100]. This technique has successfully been applied in the development of a candidate rabies vaccine encoding the main envelope glycoprotein [101].

### 6.3. Codon Usage

When designing a protein that will be expressed from an mRNA, the protein sequence needs to be converted into nucleotides. This process is not univocal, as each amino acid can be encoded by multiple codons. Sixty-one sense codons may express the 20 naturally occurring amino acids. The codon composition of a construct affects translation efficiency, mRNA abundance, and protein folding. In addition, the result is dependent on the organism and the specific cell type(s) or tissue in which the mRNA is expressed [102]. The composition of codons not only influences translation but also affects the secondary structure of mRNA, with constructs with a higher GC content being translated more efficiently than sequences characterized by a lower GC content [103]. For instance, viruses that replicate into different tissues use different synonymous codons. Codon variation affects the efficiency in which ORFs are translated across human tissues [104]. Strategies for codon optimization involve modifying codon usage to match more common tRNA species, substituting rare codons with frequent ones to expedite translation, considering neighboring nucleotides and codons to enhance the translational elongation rate [105].

However, rapid translation increases the risk of protein misfolding, potentially hampering antigen production and/or causing antigen accumulation within the cell if translation occurs too rapidly [106]. Conversely, reduced translation efficiency due to infrequently or rarely used codons can lead to the premature termination of translation that hinder protein production [107]. Codon usage and distribution throughout ORFs play a crucial role. For instance, optimizing beta-galactosidase codon usage according to the most frequently used codons for seven major proteins of a cow’s mammary cells resulted in the enhanced expression of the lacZ reporter, especially in the mammary gland [108]. The reverse is also true: codon pair deoptimization may lead to a live-attenuated virus vaccine that is apathogenic and capable of preventing infection [109]. In EBV, codon usage was found to differ between the latent and lytic infectious cycles, thus impacting disease manifestations [110].

Different methods have been used to explore the vast range of codon combinations. Zhang et al. [18] adapted the classical concepts of lattice parsing in computational linguistics to develop LinearDesign, an in silico optimization algorithm that allows for the optimal mRNA to be selected among a vast space of candidates. By defining the mRNA design space using deterministic finite-state automaton (DFA), the approach selects a vast number of mRNA candidates and allows for a number of solutions to be explored to identify the one providing the most stable molecules. This method has been applied to optimize the production of SARS-CoV-2 Spike protein, improving both mRNA half-life and protein expression. Jain and colleagues [111] introduced ICOR (Improving Codon Optimization with RNNs), a codon optimization tool that leverages deep learning and recurrent neural networks (RNNs) to enhance heterologous expression in synthetic genes. Specifically, the approach employs Bidirectional Long Short-Term Memory (BiLSTM) to learn codon usage biases alongside the contextual information surrounding codons. The algorithm was trained with a non-redundant dataset of genes derived from E. coli genomes, ensuring robustness and relevance among diverse bacterial strains. By understanding the patterns and subsequences in which synonymous codons are employed within genes, ICOR achieves a predictive accuracy that outperforms traditional methods. 

To advance development in mRNA-based medications, AI and advanced high-throughput technologies are essential. These tools play a crucial role in designing, selecting, and validating multiple aspects, such as the DNA template, sequence composition, protein structure, chemical modifications, formulation, delivery systems, and manufacturing process. The optimization of these factors impacts mRNA translation, thermostability, cellular uptake, targeting to specific organs, local expression, biodistribution, toxicity, and reactogenicity.

### 6.4. Self-Amplifying RNA Vaccines (saRNA)

Supposedly, self-amplifying RNA vaccines may offer important advantages over conventional mRNA vaccines [112,113]. First, since saRNA vaccines contain a built-in RNA polymerase, their dose can be significantly reduced. When comparing an mRNA vaccine and an saRNA vaccine in mice, saRNA preparations could achieve equivalent levels of protection against influenza virus with just 1/64th the dose. Second, saRNA vaccines are expected to induce a more durable immune response since the RNA keeps copying itself and remains in the body for prolonged times. In fact, while mRNA might last for a few days, saRNA vaccines could persist for over a month. The biodistribution of saRNA vaccines has recently been reviewed [114]. Of course, self-replicating nucleic acids could also entail safety problems linked to mutational changes during replication [115] and—theoretically—to the possible conversion of the RNA construct into cDNA followed by integration into the host genome [116].

### 6.5. Circular mRNA Constructs (circmRNA)

Circular RNAs (circmRNAs) are covalently closed circular RNA molecules synthesized by eukaryotic cells, including humans [117]. These molecules are formed through a noncanonical RNA splicing process known as backsplicing [118]. Initially considered byproducts of RNA synthesis, it is increasingly evident that they play essential roles in countless processes [119]. In comparison to linear mRNA, circmRNA is highly stable due to its closed-ring structure, shielding it from exonuclease-mediated degradation and resulting in a median half-life that is more than double that of its linear counterparts [120]. Recent studies revealed the capability of natural circmRNAs to encode and translate proteins and peptides [121]. Moreover, synthetic circmRNA constructs can be engineered to contain an IRES to initiate protein translation even in the absence of a free 5′ end [122]. Due to their distinctive properties, circmRNAs hold promise as a technology for vaccine development, particularly considering their superior stability and the high-level expression of encoded proteins [120]. Current mRNA vaccines necessitate strict storage and transportation conditions, whereas circmRNA vaccines, when encapsulated in lipid nanoparticles, can remain well preserved for at least 4 weeks at 4 °C and approximately 2 weeks at room temperature [123]. However, further research on the safety of circmRNA technology is needed before these vaccines may progress to clinical trials.

## 7. Conclusions

After mucosal immunization, essential indicators of vaccine efficacy would be levels of S-IgA in mucosal secretions, serum antibodies, and the typing of immune cells into mucosal tissue [124]. Yet, the methods devised for experimental animals cannot be directly transferred to humans. For instance, the uneven distribution and low levels of mucosal antibodies pose significant sampling and detection challenges. Existing serologic assays may not meet the sensitivity requisites for S-IgA, which is a standard for evaluating immunity in mucosae. Here, calibration reagents and methodological harmonization are still missing.

As seen during the last pandemic, the duration of immune responses remains a major challenge [15]. Typing lymphoid cell responses in tissues currently requires invasive methods that may only be used in animals: B cells in the germinal centers of lymph nodes, memory B cells and long-living plasma cells in the spleen, and memory T cells in the lamina propria of mucosae. Standardized methods to evaluate post-vaccination immunity are needed and would strongly benefit the development of vaccines capable of providing long-lasting protection.

## 8. Future Directions

The proposal of mucosal mRNA vaccines carried within EVs obtained from edible plants appears viable since—as suggested by our experience with animals—no adjuvants are needed, no safety concerns emerged from the experimental studies, and the mucosal response to a viral vaccine has been satisfactory both in the digestive and respiratory tracts. This is in line with recent results in parenterally primed animals that demonstrate the efficacy of intranasal immunization with a non-adjuvanted virus protein [125]. Similarly, recent data on humans indicate that combinations of parenteral and mucosal vaccinations can both provide systemic protection and diminish the interhuman transmission of microbial agents [29].

Finally, despite its favorable qualities, mRNA remains a vehicle, and we cannot expect a single tool to solve the problems of all antimicrobial vaccines [126]. Think about the extraordinary success of pneumococcal vaccines based on polysaccharides [127] and the beneficial off-target effects of live rotavirus vaccines [128].

To elicit protective immunity, the correct immunogens must be selected, and produced in the lab and then formulated correctly. In addition, immunization protocols are needed to stimulate and expand the rare B and T cells that represent the effectors and the memory of immune protection at different anatomical sites. Much research is needed, but the direction of future research has been set [129].
